# Designing Plant-Based Foods: Biopolymer Gelation for Enhanced Texture and Functionality

**DOI:** 10.3390/foods14091645

**Published:** 2025-05-07

**Authors:** Luísa Ozorio, Aline Beatriz Soares Passerini, Ana Paula Corradi da Silva, Anna Rafaela Cavalcante Braga, Fabiana Perrechil

**Affiliations:** 1 Department of Chemical Engineering, Universidade Federal de São Paulo (UNIFESP), Diadema 09913-030, São Paulo, Brazil; luisa.ozorio@unifesp.br (L.O.); a.passerini@unifesp.br (A.B.S.P.); ana.corradi@unifesp.br (A.P.C.d.S.); fabiana.perrechil@unifesp.br (F.P.); 2 Department of Biosciences, Universidade Federal de São Paulo (UNIFESP), Santos 11015-020, São Paulo, Brazil

**Keywords:** plant protein, pea protein, polysaccharide, gels, gellan gum

## Abstract

Despite the wide variety of plant-based products, developing high-protein products with a desirable texture remains a key challenge for the food industry. Polysaccharide and plant-protein gels offer a cost-effective strategy for meeting the growing demands of vegan and vegetarian markets. This study aimed to develop mixed pea protein–polysaccharide gels with tailored textural properties for plant-based products. The gels were prepared using pea protein and different polysaccharides, including low-acyl gellan gum (GGLA), carrageenan (CA), pectin (PEC), and high-acyl gellan gum (GGHA), along with 60 mM NaCl or CaCl_2_. The dispersions were heated to 80 °C for 30 min under mechanical stirring, followed by a pH adjustment to 7.0 with NaOH (0.1 M). The samples were then analyzed via oscillatory temperature sweep rheometry, confocal microscopy, and uniaxial compression. Self-supporting and non-self-supporting gels were obtained from the various formulations, comprising pure polysaccharide and mixed gels with diverse textures for food applications. The developed gels show a strong potential for use in meat analogs, cheeses, cream cheeses, and sauces, offering the flexibility to fine-tune their mechanical and sensory properties based on the product requirements. Combining biopolymers enables customized texture and functionality, addressing critical gaps in plant-based food innovation.

## 1. Introduction

The development of plant-based products, such as meat analogs, is a growing trend in the food sector, driven by their potential health benefits and the need to reduce environmental impacts. Another crucial aspect is the potential contribution of plant-based products to global food security. With an increase in the world population, the demand for food is growing, and conventional meat production will not be sustainable in the long term. According to a study by The Good Food Institute [1], 2.3 billion people (29.3%) worldwide face moderate or severe food insecurity, and 9.8% suffer from hunger. Therefore, plant-based products could offer a more efficient and sustainable alternative using fewer resources.

Regarding their composition, plant-based food tends to be lower in saturated fats and cholesterol and rich in fiber, vitamins, and antioxidants, promoting a more balanced diet and reducing the risk of chronic diseases, such as cardiovascular disease and diabetes [2]. However, it is worth noting that not all plant-based products are necessarily healthy. Plant-based analogs frequently have a lower protein content than the corresponding meat product [3]. Moreover, some may contain high sodium levels, additives, and preservatives, so evaluating their nutritional composition is essential.

In addition to the nutritional aspect, another challenge in creating meat analogs is reproducing the structure of animal meat with regard to its sensory properties, including texture challenges. To overcome these issues, several strategies have emerged in the literature, including extrusion, electrospinning, and 3D printing techniques associated with using biopolymers and their combinations, allowing for the creation of products with aligned fibers that mimic the texture of shredded or chunked meat. In addition, the incorporation of vegetable fats, such as coconut and sunflower oils, contributes to the sensation of the juiciness of the products and improves their sensory profiles [4].

Among the methodologies to improve plant-based food’s sensory experience, the gelation of biopolymers is a possible alternative for creating textures and sensory properties that imitate those of animal meat, which can provide an attractive consumption experience for consumers and lower production costs. Biopolymers, such as plant proteins (soy, pea, and rice) and polysaccharides (gellan gum, carrageenan, and pectin), can be used as structuring and gelling agents in vegetable meat formulations. These ingredients can form three-dimensional networks by interacting with water and other components, creating a firm and juicy texture similar to that of meat when subjected to specific pH, temperature, and ionic strength conditions. The combination of different biopolymers allows for adjustments to the mechanical and sensory properties of products, making them closer to products of animal origin, in terms of their chewiness, juiciness, and appearance [5].

Considering the challenges mentioned related to alternative plant-based food, the main objective of this study was to develop a high-protein gel matrix with different texture profiles using mixed pea protein gels with different polysaccharides, providing rich options for application in a wide variety of plant-based products, such as meat analogs, milk alternatives, cheese, and pie fillings [6,7,8,9,10].

## 2. Materials and Methods

### 2.1. Materials

The ingredients used to prepare the systems were pea protein concentrate (PE) (Pea Standard 80: 82.8% protein, 1.2% carbohydrates, 4.4% fiber, 1.8% total fat, and ≤8% ash) supplied by Gramkow Ltda (Joinville, SC, Brazil), and low-acyl gellan gum (GGLA), high-acyl gellan gum (GGHA), low-methyl pectin (PEC), and carrageenan (CA), which were kindly provided by CP Kelco (São Paulo, SP, Brazil). NaCl and CaCl_2_ used in this study were purchased from LabSynth (Diadema, SP, Brazil). All reagents employed were of analytical grade and were used without additional purification.

### 2.2. Preparation of the Gels

The samples were prepared from 15% pea protein combinations with 1% polysaccharides. CA, GGHA, GGLA, and PEC were used as polysaccharides, with and without the presence of sodium chloride (NaCl) or calcium chloride (CaCl_2_), to understand how these components interact and impact the characteristics of the gels obtained. Initially, gels containing protein and pure polysaccharides were evaluated, and in the second stage, protein combinations with polysaccharide mixtures were evaluated. The experimental samples produced are described in Table 1.

Gels were prepared according to the methodology described by Silva et al. [11]. The mixed dispersions were subjected to heat treatment at 80 °C under mechanical stirring at 500 rpm. After 30 min, the pH was adjusted to 7.0 with 0.1 M NaOH. Then, samples were immediately collected for rheological measurements and confocal microscopy. In addition, aliquots were placed in cylindrical molds and left to rest in the refrigerator at 4 °C overnight for gel formation. Molds 15 mm in diameter and 15 mm in height were used to prepare the gels to analyze the uniaxial compression. In comparison, cylinders 15 mm in diameter and 10 mm in height were used for the syneresis experiment.

### 2.3. Confocal Laser Scanning Microscopy (CLSM)

After heat treatment, proteins were stained by adding 0.5% (*v*/*v*) Rhodamine B solution (4% *m*/*v*). The stained dispersions were placed on microscope slides, covered with coverslips, and sealed with a layer of nail polish to prevent drying [12]. Samples were kept at 4 °C for at least 24 h before analysis. A Leica TCS SP8 AOBS Tandem Scanner microscope with a wavelength of 568 nm was used to excite Rhodamine-labeled proteins and analyze the mixed gels.

### 2.4. Uniaxial Compression

The tests were performed on a CT3 texture analyzer (Brookfield Engineering, Middleboro, MA, USA) coupled to a 50 mm cylindrical plate. The uniaxial compression analysis was performed in quintuplicate, according to Picone and Cunha [13], in which the equipment was configured for a single cycle of 80% deformation at 1 mm·s^−1^. The Hencky stress ($\sigma_{H}$) and Hencky strain ($\varepsilon_{H}$) were calculated according to Equations (1) and (2), while Young’s modulus is the slope of the linear part of the stress–strain curve. The stress and strain at fracture were obtained from the maximum point of the stress–strain curve.

(1) $$\sigma_{H}=\frac{F\left(t\right)\cdot h\left(t\right)}{h_{0}\cdot A_{0}}$$

(2) $$\varepsilon_{H}=-ln\left(\frac{h\left(t\right)}{h_{0}}\right)$$

where F(t) is the force at time t (N), A_0_ is the initial cross-sectional area of the samples (m^2^), h_0_ is the initial height of the sample (m), and h(t) is the height of the sample at time t.

### 2.5. Syneresis Determination

Syneresis was measured using gels of approximately 1 g, placed between two quantitative filter papers for 1 h at room temperature [14]. The gels were weighed before and after this period, and water retention was calculated according to Equation (3). The assay was performed in quintuplicate.

water retention (%) = (wf × 100)/wi,(3)

where wf is the mass of the gel after the syneresis test (g), and wi is the initial mass of the gel (g).

### 2.6. Oscillatory Rheology

Rheological measurements were performed on an MCR 92 Anton Paar GmbH rheometer (Graz, Austria) with a 50 mm parallel-plate geometry. The experiments were performed as described by Picone and Cunha [13]. Immediately after heat treatment, the dispersions were transferred to the rheometer plate, which was preheated to 80 °C. A thin layer of silicone oil was applied to the plates to prevent sample evaporation. A pre-shear at 100 s^−1^ was performed for 2 min, followed by cooling from 80 °C to 20 °C at 1 °C·min^−1^, 1% strain, and 1 Hz. Complex viscosity vs. temperature curves were used to verify the development of the gelation process. Tan δ (G″/G′) was used to evaluate the contribution of the elastic (G′) and viscous (G″) moduli during gelation.

### 2.7. Ultrafreezing and Thawing

Selected gels were subjected to an ultrafreezing process. For this, gels of approximately 1 g were taken directly to the ultrafreezer inside the molds, where they remained for 24 h. After 24 h, the gels were removed, and 5 samples were placed between two quantitative paper filters for 1 h at room temperature [14]. For the remaining 5 samples, uniaxial compression tests were performed. The masses of the gels before and after the freezing process were determined, and the water retention was calculated according to Equation (3).

### 2.8. Statistical Analysis

The results were subjected to a one-way analysis of variance (ANOVA) followed by a *t*-test using BioEstat 5.0 software. A *p*-value < 0.05 was considered statistically significant.

## 3. Results and Discussion

The gelled systems formed by plant proteins and polysaccharides present with intrinsically complex characteristics, often resulting from the synergistic interactions between the components. This complexity makes the gels highly sensitive to a wide range of experimental variables, which require rigorous control during the development and analysis of these systems.

Among the factors that directly influence the properties of the gels are the choice of protein source (PE), the polysaccharide used, the concentrations of the biopolymers, the type and charge of ions present (monovalent or divalent), the use of crosslinking agents (such as enzymes), and the pH, in addition to the process conditions, such as the temperature, preparation time, and the method for the addition of the ingredients. Polysaccharides have distinct gelation and protein interaction properties, depending upon their chemical structure and the degree of acylation or methoxylation, directly influencing the texture, opacity, and stability of the systems formed. The addition of NaCl or CaCl_2_ was explored here as a strategy to enhance or modify the molecular interactions, aiming to expand the applicability of these systems in industrial contexts, such as in the manufacture of food and biomaterials.

Due to the diversity of the objectives associated with these systems, building an initial model entirely based on the literature is not always possible, especially when focusing on specific and innovative applications. In the present study, the formulation of a meat analog was the main driver in selecting the components and experimental conditions.

### 3.1. Pure Polysaccharide Gels

**Figure 1:**
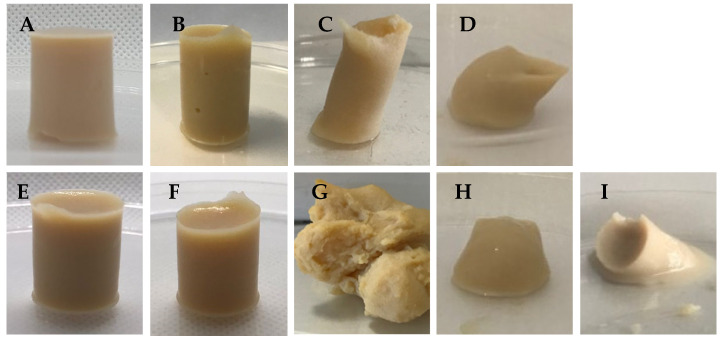
Images of gels produced using pure polysaccharides: (**A**) CA; (**B**) GGLA; (**C**) GGHA; (**D**) PEC; (**E**) CA_Na; (**F**) GGLA_Na; (**G**) GGHA_Na; (**H**) PEC_Na; and (**I**) PEC_Ca.

All the manufactured samples are shown in Figure 1, which illustrates the different gelation behaviors of the different combinations of gelling agents. The samples composed of pure polysaccharides presented different behaviors when forming gels. The formulations containing 1% CA and 1% GGLA, represented in Figure 1A,B, formed self-supporting gels characterized by the absence of visible pores. The gel obtained with 1% GGHA (Figure 1C) presented a three-dimensional structure with a low support capacity. Both forms of gellan gum were able to promote gelation. However, the native GGHA produced translucent elastic gels, while the GGLA, the most common version known for its high gelling power, produced rigid gels [15]. The sample with 1% PEC (Figure 1D) could not form a self-supporting structure under the conditions evaluated, indicating the need for strategies to improve its gel-forming capacity, considering the combination assessed in the present work.

Adding NaCl visually optimized the gel formation. Monovalent ions, such as K+, increased the interactions between the proteins and polysaccharides that helped increase the gel’s hardness and firmness [11]. In Figure 1E,F, it is possible to see that the structure with the sodium ions remained sustainable. In contrast, the samples GGHA_Na and PEC_Na, in Figure 1G,H, exhibited little support capacity.

In the specific case of the samples containing PEC, which initially did not form self-sustaining gels, the addition of CaCl_2_ was tested. The addition of CaCl_2_ promoted the formation of weak gels through interactions with the carboxylic groups of pectin (Figure 1I). In low-methoxylated pectins, gelation occurs through the formation of ionic bonds mediated by calcium ions (Ca^2+^), resulting in the formation of a continuous three-dimensional network [16,17,18].

**Figure 2:**
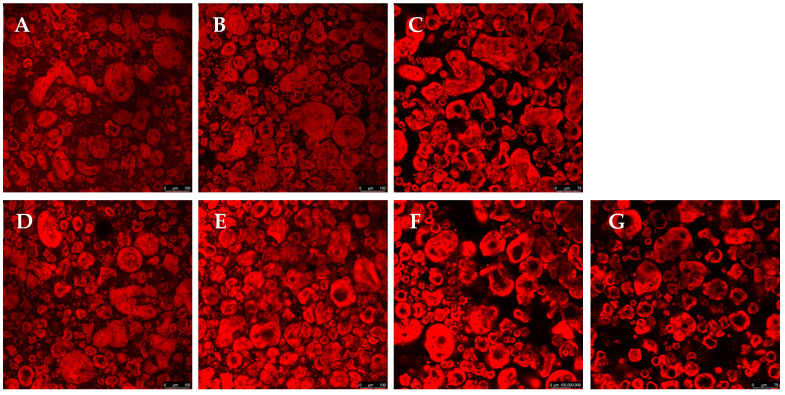
Gel morphology using confocal microscopy images for the following samples: (**A**) CA; (**B**) GGLA; (**C**) PEC; (**D**) CA_Na; (**E**) GGLA_Na; (**F**) PEC_Na; and (**G**) PEC_Ca. Scale bar = 100 µm.

Confocal microscopy allows for high-resolution three-dimensional images and is widely used to characterize complex structures in biological materials and polymers. The present study used confocal microscopy to evaluate the microstructure, phase distribution, and interaction between the components (Figure 2). The red coloration, resulting from the interaction of Rhodamine B with proteins, indicates regions of protein aggregation. At the same time, the dark areas represent polysaccharides, which do not interact with the fluorescent dye. A detailed analysis of these images allows us to understand how each component influences the structural organization of the gel. Confocal microscopy testing was not performed for the GGHA samples due to their rapid gelation.

In Figure 2A–C, for the samples containing only pea protein and polysaccharides, a relatively homogeneous distribution of the red regions is observed. This suggests that, in the absence of salt or crosslinking agents, the proteins present in the system remained more dispersed without forming significant aggregates. The morphology obtained reflects a lower interaction between the proteins and polysaccharides, resulting in a less structured network.

The addition of NaCl or CaCl_2_ to the formulations significantly altered the morphology of the gels. In Figure 2D,E, an increase in protein aggregation is observed, forming more intensely red regions. This effect can be attributed to the ability of NaCl to modify the electrostatic interactions between proteins, promoting the aggregation and stabilization of the gel. Some studies have indicated that adding NaCl before heat treatment can facilitate protein unfolding, strengthening hydrophobic interactions and leading to the formation of protein aggregates [19].

The confocal microscopy images also revealed that the composition of the gel directly affected its morphology, with significant impacts from the type of polysaccharide on the structural organization of the gels. While the gelled systems with CA and GGLA showed a more homogeneous structure, the gels with PEC showed a micro-phase separation, with a clear formation of protein-rich domains dispersed on a continuous polysaccharide phase [20]. This phenomenon was observed even with salt addition. Phase separation is a common phenomenon that occurs in mixtures of proteins and polysaccharides due to the thermodynamic incompatibility and repulsion between biopolymers with the same charge. However, when the gelation process is fast, it immobilizes the structure, resulting in a micro-phase separation in the gel [21,22].

Based on these results, only the formulations containing CA and GGLA, with or without NaCl, were selected for evaluation by uniaxial compression, syneresis, and rheological analyses, as they were the only samples that formed self-supporting structures.

**Figure 3:**
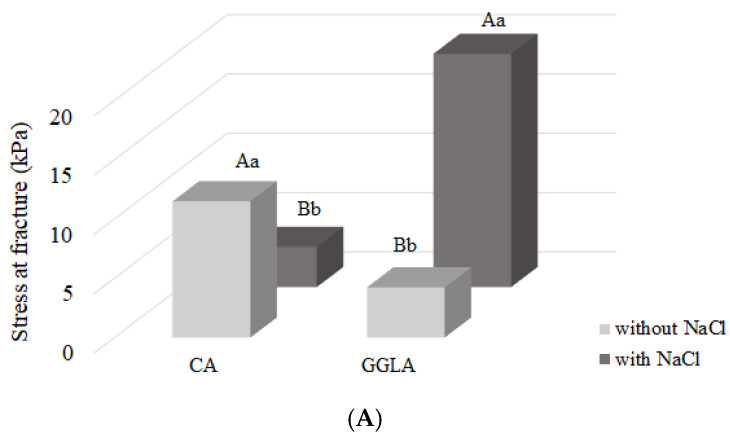

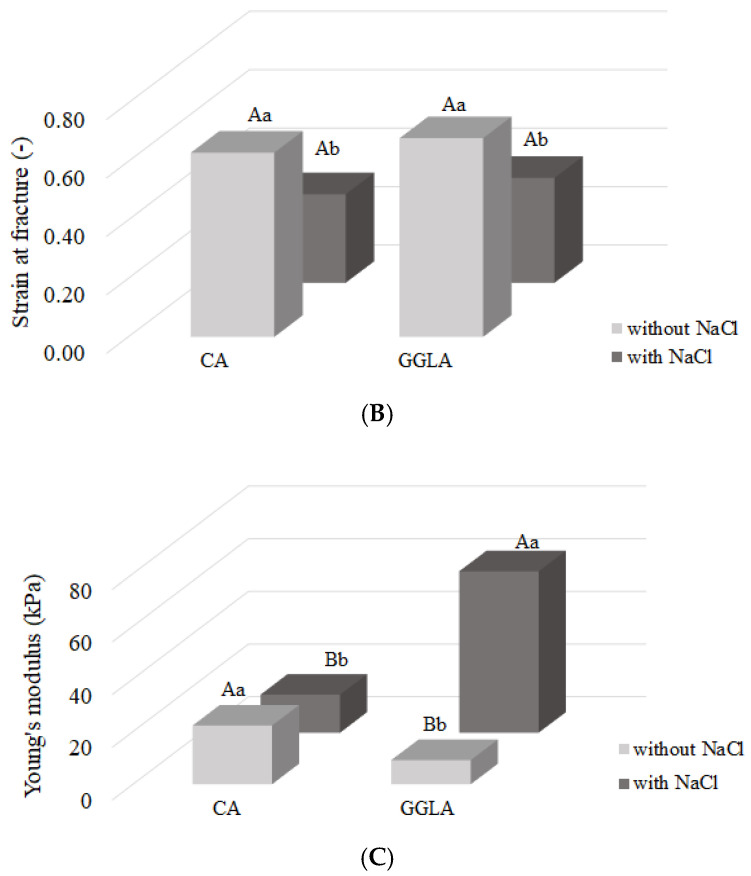
Mechanical properties of pea protein and pure polysaccharide (CA or GGLA) gels with the presence or absence of NaCl: (**A**) stress at fracture; (**B**) strain at fracture; and (**C**) Young’s modulus. Uppercase letters indicate significant differences between different polysaccharides at the same salt concentration, while lowercase letters indicate differences within the same polysaccharide, with and without salt (*p* < 0.05).

To respond to the panoramic texture behavior of the produced gels, the analysis of the mechanical behavior during uniaxial compression was evaluated through the stress at fracture (Figure 3A), strain at fracture (Figure 3B), and Young’s modulus (Figure 3C). All the experimental conditions in the graphs refer to the concentrations described in Table 2, ensuring consistency between the data presented and the parameters established during the research.

From Figure 3A, it is possible to observe distinct behaviors among the analyzed gels with different polysaccharides (CA or GGLA) regarding the fracture stress in the presence or absence of NaCl. For CA, it is observed that the lack of sodium ions resulted in a significant increase in the fracture stress, reaching 19.68 ± 1.78 kPa, compared to the sample containing NaCl, which reached 3.39 ± 0.68 kPa. This difference of 5.81 times shows that CA displays a better mechanical performance without salt. Therefore, the addition of sodium ions can negatively interfere with the formation and stabilization of the gel network. The same behavior was observed by Ipsen [23], who investigated systems composed of pea protein isolate (PEI) or soy protein isolate (PSI) in combination with κ-carrageenan in the presence of calcium ions or sodium chloride. The study demonstrated that adding NaCl significantly reduced the strength and stiffness of the gels, regardless of the protein source, which was attributed to the low solubility of CA in the presence of salt, not achieving the formation of an optimal gel network. In addition, CA is found in the coil state at room temperature in the presence of Na^+^, making its gelation difficult [24].

On the other hand, GGLA showed the opposite behavior. The presence of NaCl resulted in a higher rupture voltage, reaching 11.50 ± 1.01 kPa, compared to the sample without salt, which reached 4.24 ± 0.60 kPa. This 2.71-fold increase indicates that sodium ions favor the formation of a harder gel network. Sodium ions act by facilitating interactions between polymer chains, promoting the greater organization and resistance of a gel network. This occurs because monovalent cations act as a shield against the electrostatic repulsion between gellan chains, facilitating the gelling of gellan gum [25].

When analyzing Figure 3B, it is observed that both CA and GGLA present with a higher fracture strain in the absence of sodium ions, respectively, of 0.63 ± 0.07 and 0.68 ± 0.09 (*p* > 0.05). This behavior can be explained by the influence of monovalent cations, such as Na^+^, in the structuring of the gels. Both CA and GGLA have similar gelation mechanisms, with a coil-to-helix transition, followed by the self-assembling of helices to produce the gels [13,26]. However, while Na^+^ ions make the coil-to-helix transition difficult for CA [24], they increase the assembling of GGLA helices [27]. As a result, the addition of NaCl results in weaker and less deformable gels for CA, due to the fragility of the gel network, and harder and less deformable gels for GGLA, associated with the strengthening of the structure of the junction zones.

It is known that Young’s modulus reflects a material’s resistance to small deformations and indicates the firmness of the gel. On the other hand, the fracture stress is associated with the hardness of a material [28]. In many cases, Young’s modulus and the stress at fracture exhibit similar behaviors, especially when the variation in a variable, such as the concentration, is analyzed. However, this trend is not a rule. In Figure 3C, the Young’s modulus results show a behavior similar to that observed for the fracture stress, a pattern that, as reported by Silva et al. [11], is common in gels. The CA without salt showed an increase of 1.55 kPa compared to the sample containing salt, while the GGLA without salt demonstrated a more impressive increase of 52.16 kPa.

**Figure 4:**
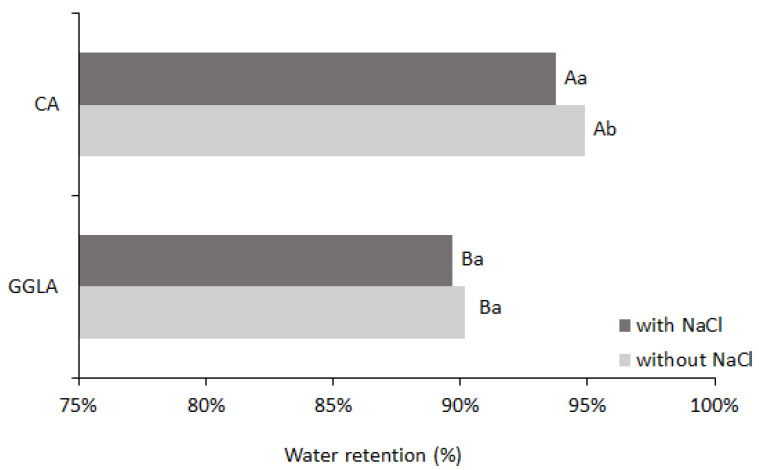
Water retention after syneresis analysis for pea protein and pure polysaccharide (CA or GGLA) gels with or without NaCl. Uppercase letters indicate significant differences between different polysaccharides at the same salt concentration, while lowercase letters indicate differences within the same polysaccharide, with and without salt (*p* < 0.05).

Syneresis is often considered an undesirable characteristic, which negatively affects the quality of food products [29]. Therefore, a gel should have a good water retention capacity, contributing to its greater stability over time and during the processes it goes through, such as cooking. In addition, water retention also has a positive impact on the juiciness of the gelled product [11]. Figure 4 shows the percentage of water retention after the syneresis analysis.

Analyzing Figure 4, the sample with CA presented with higher water retention values (94.87% without sodium ions and 93.77% with sodium ions) than the gels with GGLA (90.16% without sodium ions and 89.66% with sodium ions). The excellent water-holding capacity of CA gels has already been reported in the literature [30,31], and may be attributed to its chemical structure, which is rich in sulfated groups, favoring electrostatic interactions and increasing its affinity for water. For both gels, the addition of NaCl reduced the water retention efficiency. This occurred because the sodium ions interfered in the interactions between the biopolymers and water, competing for water molecules and reducing the cohesion of the three-dimensional networks [32]. Therefore, the gels with NaCl had lower water retention than those without salt; this may be related to a salting-out effect, with the approximation of the biopolymers and the exclusion of water.

**Figure 5:**
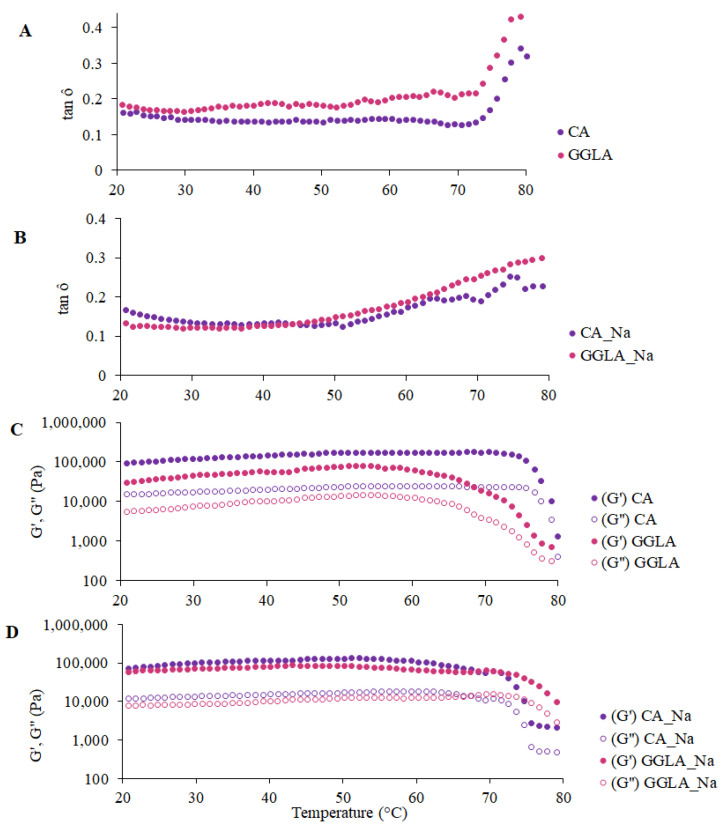
Rheological measurements for gels with pure polysaccharide and pea protein during the cooling cycle (80 °C to 20 °C): (**A**) tan δ for gels CA and GGLA; (**B**) tan δ for gels CA_Na and GGLA_Na; (**C**) G′ and G″ (Pa) for samples CA and GGLA; and (**D**) G′ and G″ (Pa) for samples CA_Na and GGLA_Na.

In terms of the rheological measurements, Figure 5 presents the tan δ and the G′ and G″ moduli for the gels with pea protein and pure polysaccharides CA and GGLA, with the presence or absence of NaCl.

The tan δ results are presented in Figure 5A,B, indicating that, since the first recording at 80 °C, all the samples presented with tan δ values lower than 1. A progressive drop in the tan δ values was observed with a reduction in the temperature, showing that the gels became firmer. The tan δ parameter is defined as the quotient between the viscous (G″) and elastic (G′) moduli and is indicative of the structural nature of a material: tan δ values < 1 reflect predominantly solid behavior, while tan δ > 1 characterizes a viscous or liquid structure [33,34]. Hence, the results confirm that gel structures had already formed in all the formulations analyzed, since the G′ was higher than the G″ (i.e., tan δ < 1) throughout the temperature range evaluated.

In Figure 5A,B, it can be seen that the gel composed of GGLA presented with the lowest tan δ values, suggesting greater structural rigidity. In addition, there was a more pronounced reduction in this parameter up to approximately 70 °C, followed by stabilization for all the samples. Previous studies, such as that of Picone and Cunha [13], evaluated gellan gum gels (1.5% *w/w*) at pH 7.0 and identified that the sol–gel transition occurred at 34.2 ± 0.6 °C. Thus, in the present study, the protein content and the addition of monovalent salt may have influenced the gelation temperature of the gellan gum, as discussed by Ozorio et al. [35].

The behavior of the elastic (G′) and viscous (G″) moduli, illustrated in Figure 5C,D, corroborates the trends observed for the tan δ. In all the samples, the G′ was consistently greater than the G″, indicating the formation of a well-structured three-dimensional network characteristic of solid gels. Gels have a semi-solid structure due to their polymer network that retains the liquid phase. Their rheological behaviors are crucial in the formulation and development of products, directly influencing the choice of the appropriate system according to the desired application [36].

Figure 5C,D also show the differences between CA and GGLA during the gelation process. In the absence of salt (Figure 5C), the gels with CA showed a fast increase in the G′ modulus, while this variable slowly increased in the GGLA gels. On the other hand, the opposite tendency was observed for the gels with NaCl. This result corroborates the results discussed earlier, which showed that Na^+^ ions hindered the formation of CA helices, while they can reinforce the self-assembly of GGLA helices during the gelation process.

In order to assess the influence of freezing as a possible process of conservation, the gels made of pea protein with pure GGLA and CA polysaccharides were subjected to subsequent performance evaluation analyses after deep freezing and thawing. The comparison of the mechanical properties (Table 2) showed that the gels became weaker and less rigid after the freezing–thawing cycle, with exception of sample GGLA that became more rigid. It is interesting to note that freezing and thawing may have caused greater weakening of the stronger gels (samples CA and GGLA_Na). In addition, the gels without NaCl were less deformable after the freezing–thawing cycle, while the strain at fracture did not change for the samples with NaCl.

Table 2 also shows the water retention results for the GGLA and CA gels, with and without adding NaCl, after freezing and thawing. This test was essential to evaluate the ability of the gels to retain water and resist the phenomenon of syneresis after freezing (loss of water through the gel network). The addition of NaCl led to a negative effect on the water retention for both GGLA and CA. This may have occurred because the Na^+^ ions competed with the water molecules for interactions with the polymer chains, reducing the capacity of the gel network to retain water [32].

Freezing impacts the gel structure because freezing can cause the formation of large ice crystals, which rupture the gel network, resulting in the weakening of the structure and greater water release during thawing [37].

### 3.2. Mixture of Polysaccharides Gels

**Figure 6:**
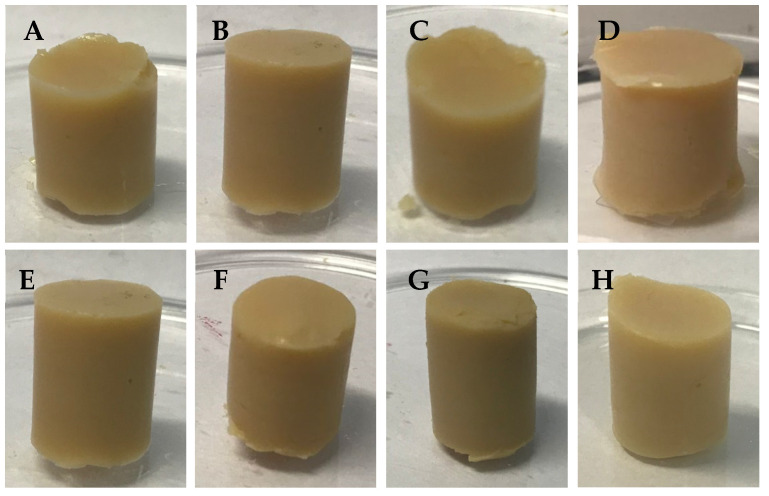
Images of gels from a mixture of polysaccharides: (**A**) CA_GGLA; (**B**) GGLA_GGHA; (**C**) CA_GGHA; (**D**) CA_GGLA_GGHA; (**E**) CA_GGLA_Na; (**F**) GGLA_GGHA_Na; (**G**) CA_GGHA_Na; and (**H**) CA_GGLA_GGHA_Na.

Since different polysaccharides can confer various properties to gels, especially in terms of texture, it was decided to analyze some mixtures of these biopolymers (Table 1) and compare the parameters evaluated for the gels of pea protein with those of pure polysaccharides. It is important to note that the concentration of GGHA was limited to 0.05%, as higher concentrations resulted in excessively high viscosity, which hindered the proper filling of the molds. Figure 6 illustrates the appearance of the protein gels with polysaccharide mixtures. The visual analysis of these samples allowed for identifying the color, translucency, shape, and structural integrity variations, which may be related to their composition and experimental conditions.

In general, the polysaccharide mixtures used allowed for the formation of self-supporting and similar gels. Compared to the pure polysaccharide samples, the mixed gels also showed visual similarity with the self-supporting protein-pure polysaccharide gels.

**Figure 7:**
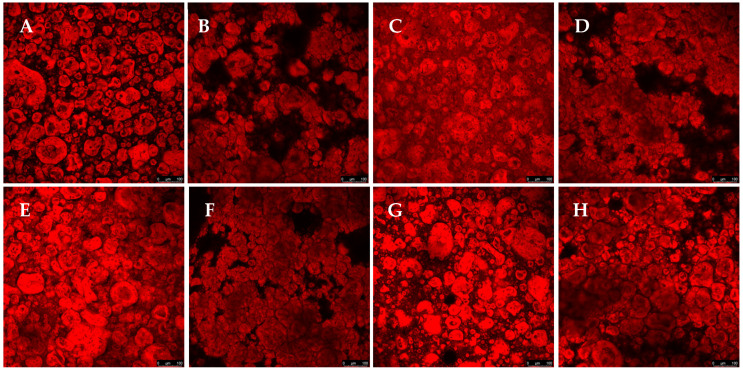
Gel morphology, using confocal microscopy images, for the samples composed by a mixture of polysaccharides: (**A**) CA_GGLA; (**B**) GGLA_GGHA; (**C**) CA_GGHA; (**D**) CA_GGLA_GGHA; (**E**) CA_GGLA_Na; (**F**) GGLA_GGHA_Na; (**G)** CA_GGHA_Na; and (**H**) CA_GGLA_GGHA_Na. Scale bar = 100 µm.

Figure 7 shows that the interaction between different polysaccharides influenced the protein phase distribution and each gel’s structural organization. In the samples without salt, it was observed that the protein aggregation varied according to the composition of the gel. When there was a predominance of gellan gum (samples GGLA_GGHA and CA_GGLA_GGHA), the protein aggregation increased, resulting in more concentrated and defined red areas with a micro-phase separation (Figure 7B,D). In the samples with CA (samples CA_GGLA and CA_GGHA), the protein appeared more soluble and homogeneously distributed (Figure 7E,G).

With the addition of NaCl, there was an increase in protein aggregation in all the samples, regardless of the polysaccharide. This occurred because the monovalent ion Na^+^ reduced the electrostatic repulsion between the proteins and polysaccharides, favoring their association and forming more aggregated structures [11]. Compared to Figure 2, CA kept the protein relatively more dispersed, even in salt’s presence, while GGLA intensified aggregation, mainly with the addition of GGHA.

**Figure 8:**
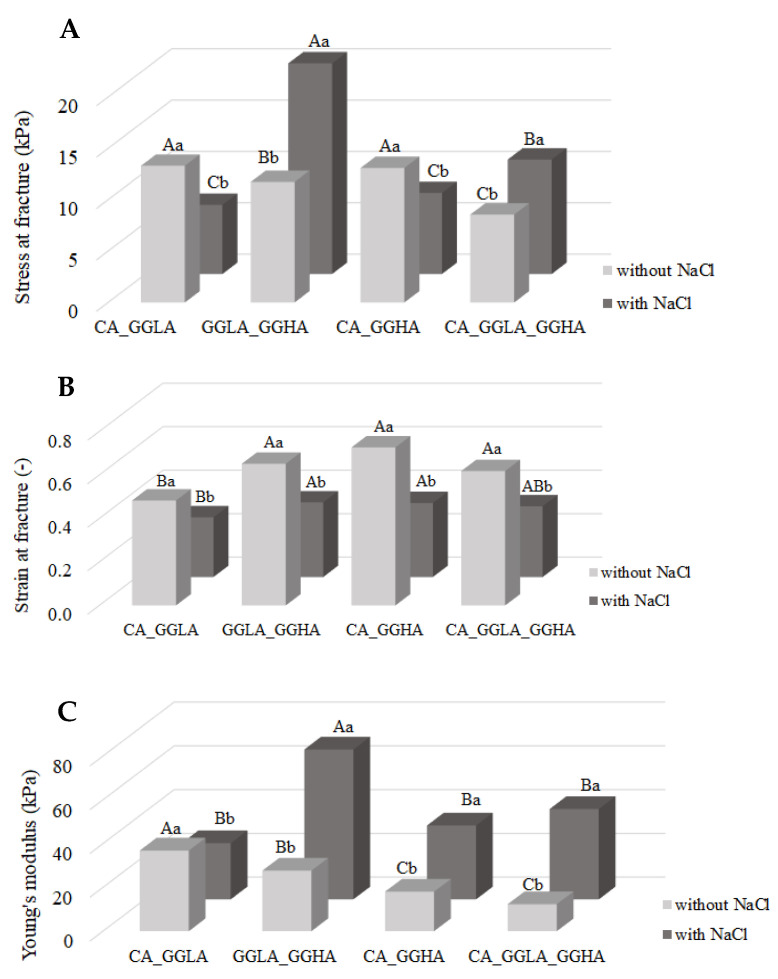
Mechanical properties of pea protein and mixtures of polysaccharides. (**A**) stress at fracture; (**B**) strain at fracture; and (**C**) Young’s modulus. Uppercase letters indicate significant differences between different polysaccharide mixtures at the same salt concentration, while lowercase letters indicate differences within the same polysaccharide mixture, with and without salt (*p* < 0.05).

The analysis of the mechanical behavior during uniaxial compression was also evaluated for the mixture samples (Figure 8) through the stress at fracture (Figure 8A), strain at fracture (Figure 8B), and Young’s modulus (Figure 8C). For the gels without salt, samples CA_GGLA and CA_GGHA showed the highest values of stress at fracture. However, the addition of salt (NaCl) reduced the fracture stress of these formulations and increased the hardness of samples GGLA_GGHA_Na and CA_GGLA_GGHA_Na. These results suggest that the influence of NaCl is directly linked to the composition and proportion of the polysaccharides in a formulation, determining the structural network’s capacity for formation and resistance. Thus, for the samples CA_GGLA and CA_GGHA, the behavior was determined by the CA, whose gels weaken in the presence of NaCl [24]. On the contrary, for the formulations GGLA_GGHA and CA_GGLA_GGHA, the behavior was determined by the gellan gum, whose gels are reinforced in the presence of monovalent ions [25], as observed in Figure 3.

It is interesting to note that the gels with a mixture of polysaccharides (Figure 3) were harder than those containing pure polysaccharides, indicating a synergistic effect among the polysaccharides in the structuring the gel network. The harder gel was observed for the sample with the higher concentration of GGLA in the presence of NaCl (GGLA_GGHA_Na), with a fracture stress reaching 20.51 kPa. This result may be related to the interaction between gellan gum and NaCl, as discussed earlier.

As shown in Figure 8B, which presents the data for the strain at fracture, the absence of sodium ions resulted in more deformable gels in all formulations. This behavior was also identified in isolated polysaccharides (Figure 3) and can be attributed to the absence of ionic interactions promoted by NaCl. This led to a less rigid and, consequently, more elastic polymer network. The presence of NaCl favored electrostatic interactions between the polymer chains, especially in the presence of gellan gum, resulting in more structured and less deformable gels. These results reinforce the role of Na^+^ ions as structuring agents, restricting the mobility of polymer chains and conferring greater rigidity to gels. On the other hand, the absence of NaCl provides greater flexibility to a polymer matrix, allowing gels to withstand a greater deformation before rupture [35,38].

This comparison of the different polysaccharide compositions shows that the samples containing GGHA presented with a greater strain at fracture when compared to the formulations without this polysaccharide. It is well known that the gels of GGHA have a lower hardness and much higher deformability than GGLA [39]. The results of the present work show that even a low concentration of GGHA (0.05%) is sufficient to increase the elasticity of gels.

Finally, in Figure 8C, it can be observed that Young’s modulus did not follow the same trend as the stress at rupture. Samples GGLA_GGHA_Na, CA_GGHA_Na, and CA_GGLA_GGHA_Na presented with higher Young’s modulus values, indicating a greater rigidity of the gels. This behavior can be attributed to the formation of stronger ionic bonds between the polysaccharides and the sodium ions, resulting in a more compact gel network resistant to deformation. The effect appears to be associated with the presence of GGHA in the samples GGLA_GGHA_Na, CA_GGHA_Na and CA_GGLA_GGHA_Na. On the other hand, the sample CA_GGLA_Na showed a behavior similar to that observed in pure polysaccharide gels, maintaining the tendency of stress at fracture. The sample GGLA_GGHA_Na showed the most significant increase in Young’s modulus (68.48 kPa) with salt addition, a result that was also reflected in the stress at fracture. This substantial increase can be attributed to the better packing of the polymer chains provided by the interaction among NaCl, GGLA, and GGHA, which intensified the intermolecular interactions and conferred greater mechanical strength to the gel.

**Figure 9:**
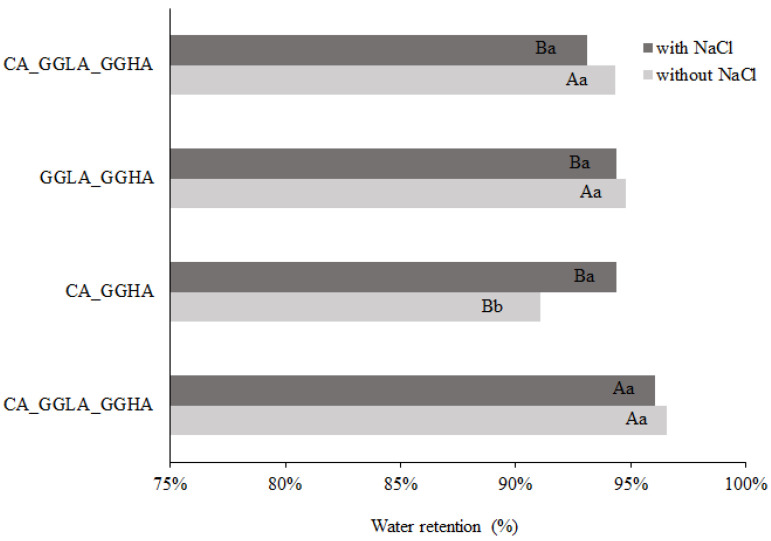
Syneresis for gels of pea protein and polysaccharide mixtures with or without the presence of NaCl. Uppercase letters indicate significant differences between different polysaccharide mixtures at the same salt concentration, while lowercase letters indicate differences within the same polysaccharide mixture, with and without salt (*p* < 0.05).

Figure 9 shows the results for water retention of systems composed of pea protein and a mixture of polysaccharides, with and without NaCl. In general, the systems did not present significant differences between the polysaccharides with and without salt. An exception was the sample rich in CA, which presented with greater water retention in the presence of NaCl. This trend was opposite to that observed for the protein-pure polysaccharide gels, which can be explained by the presence of GGHA in the formulation.

The formulation that combined all the polysaccharides (CA_GGLA_GGHA) showed the highest water retention, both in the presence and absence of salt, reaching a retention index of approximately 96%. This superior performance, compared to gels formed exclusively by protein and pure polysaccharides, can be attributed to the synergistic effect among the components, which enhanced water retention. In addition, it is possible that the specific contribution of GGHA played a relevant role, positively influencing the structure of the gel network, as previously observed by Buldo et al. [40].

On the other hand, the sample CA_GGHA showed the lowest performance when evaluated without salt, registering a water retention of only 90.69%. This behavior can be explained by the high interaction between the biopolymers, as observed in Figure 7, expelling the water from the network structure. In the presence of NaCl, this gel is weakened (Figure 8), i.e., the interactions between the biopolymers are not so strong, leading to an increase in the water retention.

This difference in performance highlights the importance of both the composition of the polysaccharides and the presence of salt for controlling the water retention capacity, evidencing how structural and interactional factors directly affect the functional properties of gel networks.

**Figure 10:**
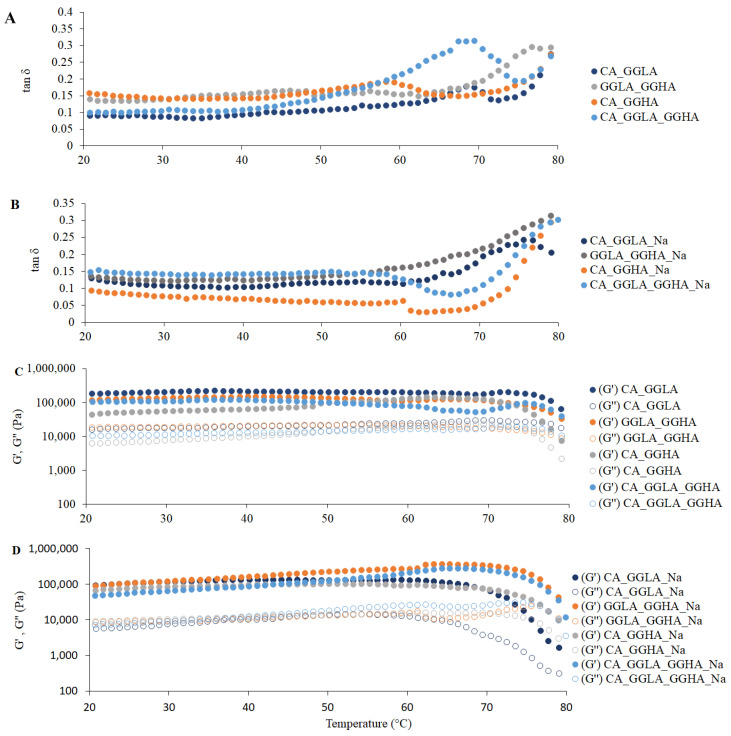
Rheological measurements for gels with pure polysaccharide and pea protein during the cooling cycle (80 °C to 20 °C): (**A**) tan δ for gels without NaCl; (**B**) tan δ for gels with NaCl; (**C**) G′ and G″ (Pa) for samples without NaCl; and (**D**) G′ and G″ (Pa) for samples with NaCl.

The results of the rheological measurements for the mixed polysaccharides (CA, GGLA, and GGHA) and pea protein with the presence or absence of NaCl are presented in Figure 10. For the samples without NaCl (Figure 10A), the tan δ was consistently lower than one throughout the temperature range (80–20 °C). This indicates that the gel structures were already formed, with predominantly solid behavior (G′ > G″). The differences among the samples CA_GGLA, GGLA_GGHA, CA_GGHA, and CA_GGLA_GGHA were associated with the concentrations of and interactions among the biopolymers, with the sample CA_GGLA presenting with the lowest tan δ value, suggesting greater structural rigidity. In the samples containing NaCl (Figure 10B), the tan δ was also less than one. Still, there was a more pronounced reduction in this parameter up to 60 °C, followed by stabilization for all the samples, and the tan δ values were initially higher at 80 °C. This indicates that the formulations with salt were initially more liquid at 80 °C, which may have facilitated the incorporation of these samples into the molds. The sample CA_GGHA_Na presented with the lowest value for this parameter, indicating that the combination of a higher concentration of CA and NaCl and a low temperature led to a more rigid gel.

In Figure 10C,D, the behavior of the elastic (G′) and viscous (G″) moduli reinforces the observations for the tan δ. The elastic modulus (G′) was consistently higher than the viscous modulus (G″), indicating the formation of a well-structured three-dimensional network characteristic of solid gels. This trend was more pronounced at room temperature in the samples without NaCl, which appeared more structured than those with NaCl, except for sample P.

## 4. Conclusions

The findings of this study demonstrate that biopolymer gelation using pea protein concentrate and polysaccharides (gellan gum, carrageenan, and pectin) offers a promising strategy for developing functional plant-based products, particularly as analogs for animal-derived foods. This is already a reality in the market (in plant-based products of global brands) as well as in the academic world, since reports in the literature have shown improvements in texture using gels in cheese, meat sausage analogs, mushroom sausage, plant-based yogurt, etc.

Combining biopolymers under varied conditions enabled the production of gels with tailored textures suitable for diverse food applications. The formulation GGLA_GGHA_Na exhibited a superior hardness, Young’s modulus, and tensile strength, making it ideal for meat analogs. Additionally, the PEC-based gels displayed softer, more fluid textures, aligning with applications like plant-based milk or cream cheese alternatives. Regarding the functional advantages, polysaccharides significantly enhanced water retention, structural stability, and the ability to mimic the chewiness and juiciness of animal-derived products.

The developed gels hold strong potential for broad use in plant-based foods, including meat analogs (e.g., burgers, sausages) and dairy alternatives (cheeses, cream cheeses, and fillings). Also, these gels’ customizable mechanical/sensory properties allow for precise adaptations to product requirements. The methodology leverages simple techniques (temperature, ionic strength, and pH control), ensuring scalability without specialized equipment. This innovation advances plant-based food technology and supports the growing demand for sustainable, nutritious alternatives, diversifying the market.

## Figures and Tables

**Table 1:** Gel compositions of pea protein samples with pure and mixture of polysaccharides.

Samples	CA	GGLA	GGHA	PEC	NaCl (mM)	CaCl_2_ (mM)
CA	1%	-	-	-	-	-
GGLA	-	1%	-	-	-	-
GGHA	-	-	1%	-	-	-
PEC	-	-	-	1%	-	-
CA_Na	1%	-	-	-	60	-
GGLA_Na	-	1%	-	-	60	-
GGHA_Na	-	-	1%	-	60	-
PEC_Na	-	-	-	1%	60	-
PEC_Ca	-	-	-	1%	-	60
CA_GGLA	0.5%	0.5%	-	-	-	-
GGLA_GGHA	-	0.95%	0.05%	-	-	-
CA_GGHA	0.95%	-	0.05%	-	-	-
CA_GGLA_GGHA	0.475%	0.475%	0.05%	-	-	-
CA_GGLA_Na	0.5%	0.5%	-	-	60	-
GGLA_GGHA_Na	-	0.95%	0.05%	-	60	-
CA_GGHA_Na	0.95%	-	0.05%	-	60	-
CA_GGLA_GGHA_Na	0.475%	0.475%	0.05%	-	60	-

All samples presented comprised 15% pea protein with 1% polysaccharides.

**Table 2:** Uniaxial compression and syneresis analyses for pea protein and pure polysaccharide (CA or GGLA) gels with the presence or absence of NaCl after ultrafreezing.

		Before Freezing		After Thawing	
		CA	GGLA	CA	GGLA
Stress at fracture (kPa)	Without NaCl	11.50 ± 1.01	4.24 ± 0.60	3.47 ± 0.41	3.91 ± 0.78
	With NaCl	3.39 ± 0.68	19.68 ± 1.78	2.72 ± 0.67	12.77 ± 1.84
Strain at fracture (kPa)	Without NaCl	0.63 ± 0.07	0.68 ± 0.09	0.33 ± 0.02	0.27 ± 0.05
	With NaCl	0.30 ± 0.07	0.36 ± 0.03	0.32 ± 0.04	0.34 ± 0.03
Young’s modulus (kPa)	Without NaCl	22.30 ± 3.94	9.17 ± 0.53	12.23 ± 3.46	14.98 ± 3.49
	With NaCl	14.48 ± 2.67	61.33 ± 12.31	4.94 ± 0.80	32.91 ± 2.85
Water retention (%)	Without NaCl	94.87 ± 0.75	90.21 ± 0.70	94.41 ± 0.41	87.23 ± 0.78
	With NaCl	93.77 ± 0.70	89.66 ± 3.39	84.22 ± 0.67	81.30 ± 1.84

## Data Availability

The original contributions presented in the study are included in the article, further inquiries can be directed to the corresponding author.

## References

[1] Lupetti C., Casselli R. (2022). O Consumidor Brasileiro e o Mercado Plant-Based.

[2] El Sadig R., Wu J. (2024). Are Novel Plant-Based Meat Alternatives the Healthier Choice?. Food Res. Int..

[3] Locatelli N.T., Chen G.F.N., Batista M.F., Furlan J.M., Wagner R., Bandoni D.H., de Rosso V.V. (2024). Nutrition Classification Schemes for Plant-Based Meat Analogues: Drivers to Assess Nutritional Quality and Identity Profile. Curr. Res. Food Sci..

[4] Sha L., Xiong Y.L. (2020). Plant Protein-Based Alternatives of Reconstructed Meat: Science, Technology, and Challenges. Trends Food Sci. Technol..

[5] McClements D.J., Grossmann L. (2021). The Science of Plant-Based Foods: Constructing next-Generation Meat, Fish, Milk, and Egg Analogs. Compr. Rev. Food Sci. Food Saf..

[6] Nie Y., Xiong Y.L., Jiang J. (2025). Texture, Microstructure, and in Vitro Digestion of Hybrid Meat Gel-Type Sausages Formulated with Functionalized Pea Protein. Food Hydrocoll..

[7] Ghimire S., Umar M., Gamonpilas C., Anal A.K. (2025). Improving Rheology and 3D Printability of Pea, Fava and Mung Bean Proteins with Raw and Fermented Millet Flour. Food Hydrocoll..

[8] Ren W., Liang H., Li B., Li J. (2025). Towards Ideal Plant-Based Yogurts: Evaluating Component and Processing Effects on Mouthfeel and Stability. J. Future Foods.

[9] Sim J.H., Moon S., Kim J.H., Lee C., Yu D. (2025). Influence of Plant-Based Gel Binders and Song-Hwa Mushroom Crosslinking on Functional Properties and Consumer Perception of Vegan Mushroom Sausage Analogues. Food Chem..

[10] Jian C., Yang X., Tuccillo F., Hashim M., Cera S., Yan J.-K., Coda R., Maina N.H., Katina K., Wang Y. (2025). Impact of Fermentation Conditions and Dextran Structure on the Rheological and Textural Properties of a Novel High-Protein, High-Fiber and Low-Fat Plant-Based Cheese. Food Hydrocoll..

[11] Silva F.G., Passerini A.B.S., Ozorio L., Picone C.S.F., Perrechil F.A. (2024). Interactions between Pea Protein and Gellan Gum for the Development of Plant-Based Structures. Int. J. Biol. Macromol..

[12] Perrechil F.A., Braga A.L.M., Cunha R.L. (2009). Interactions between Sodium Caseinate and LBG in Acidified Systems: Rheology and Phase Behavior. Food Hydrocoll..

[13] Picone C.S.F., Cunha R.L. (2011). Influence of PH on Formation and Properties of Gellan Gels. Carbohydr. Polym..

[14] Pires Vilela J.A., Cavallieri Â.L.F., Lopes da Cunha R. (2011). The Influence of Gelation Rate on the Physical Properties/Structure of Salt-Induced Gels of Soy Protein Isolate–Gellan Gum. Food Hydrocoll..

[15] Ng J.Y., Obuobi S., Chua M.L., Zhang C., Hong S., Kumar Y., Gokhale R., Ee P.L.R. (2020). Biomimicry of Microbial Polysaccharide Hydrogels for Tissue Engineering and Regenerative Medicine—A Review. Carbohydr. Polym..

[16] Han W., Meng Y., Hu C., Dong G., Qu Y., Deng H., Guo Y. (2017). Mathematical Model of Ca2+ Concentration, PH, Pectin Concentration and Soluble Solids (Sucrose) on the Gelation of Low Methoxyl Pectin. Food Hydrocoll..

[17] Cao L., Lu W., Mata A., Nishinari K., Fang Y. (2020). Egg-Box Model-Based Gelation of Alginate and Pectin: A Review. Carbohydr. Polym..

[18] Said N.S., Olawuyi I.F., Lee W.Y. (2023). Pectin Hydrogels: Gel-Forming Behaviors, Mechanisms, and Food Applications. Gels.

[19] Zhang Y., Herneke A., Langton M., Johansson M., Corredig M. (2025). Effect of PH and Ionic Strength on Heat-Induced Pea Protein Isolate Aggregation and Gel Formation. Food Hydrocoll..

[20] Ako K., Durand D., Nicolai T. (2011). Phase Separation Driven by Aggregation Can Be Reversed by Elasticity in Gelling Mixtures of Polysaccharides and Proteins. Soft Matter.

[21] Yang Y., Yu J., Ding Q., Qian S. (2025). Micro-Phase Separation and Rheological Behavior of Whey Protein Isolate/Gellan Gum Mixed Gels. Food Hydrocoll..

[22] de Jong S., van de Velde F. (2007). Charge Density of Polysaccharide Controls Microstructure and Large Deformation Properties of Mixed Gels. Food Hydrocoll..

[23] Ipsen R. (1997). Uniaxial Compression of Gels Made from Protein and K-Carrageenan. J. Texture Stud..

[24] Mangione M.R., Giacomazza D., Bulone D., Martorana V., Cavallaro G., San Biagio P.L. (2005). K+ and Na+ Effects on the Gelation Properties of κ-Carrageenan. Biophys. Chem..

[25] Huang Y., Yang N., Zhang Y., Hou J., Gao Y., Tian L., Jin Z., Shen Y., Guo S. (2021). The Gelling Behavior of Gellan in the Presence of Different Sodium Salts. Int. J. Biol. Macromol..

[26] Hilliou L. (2021). Structure–Elastic Properties Relationships in Gelling Carrageenans. Polymers.

[27] Moritaka H., Fukuba H., Kumeno K., Nakahama N., Nishinari K. (1991). Effect of Monovalent and Divalent Cations on the Rheological Properties of Gellan Gels. Food Hydrocoll..

[28] Guo J., Liu Y.C., Yang X.Q., Jin Y.C., Yu S.J., Wang J.M., Hou J.J., Yin S.W. (2014). Fabrication of Edible Gellan Gum/Soy Protein Ionic-Covalent Entanglement Gels with Diverse Mechanical and Oral Processing Properties. Food Res. Int..

[29] Rafiq L., Zahoor T., Sagheer A., Khalid N., Ur Rahman U., Liaqat A. (2019). Augmenting Yogurt Quality Attributes through Hydrocolloidal Gums. Asian-Australas. J. Anim. Sci..

[30] Saha D., Bhattacharya S. (2010). Hydrocolloids as Thickening and Gelling Agents in Food: A Critical Review. J. Food Sci. Technol..

[31] Lu Z., Lee P.R., Yang H. (2023). Kappa-Carrageenan Improves the Gelation and Structures of Soy Protein Isolate through the Formation of Hydrogen Bonding and Electrostatic Interactions. Food Hydrocoll..

[32] Joshi S.C. (2011). Sol-Gel Behavior of Hydroxypropyl Methylcellulose (HPMC) in Ionic Media Including Drug Release. Materials.

[33] Thim L., Madsen F., Poulsen S.S. (2002). Effect of Trefoil Factors on the Viscoelastic Properties of Mucus Gels. Eur. J. Clin. Investig..

[34] Shevkani K., Singh N., Kaur A., Rana J.C. (2015). Structural and Functional Characterization of Kidney Bean and Field Pea Protein Isolates: A Comparative Study. Food Hydrocoll..

[35] Ozorio L., Corradi A.P., Perrechil F. (2024). Pea and Rice Proteins with Gellan Gum and Monovalent Salts for the Development of Plant-Based Gels for Food Applications. Appl. Food Res..

[36] Reolon J.B., Hammerschmitt B.K., Sari M.H.M., Lazo R.E.L., de Fátima Cobre A., Capeletti M.B., Rigo M., Bonini J.S., da Rosa Abaide A., Pontarolo R. (2024). Predictive Modeling of Rheological Behavior in Semisolid Pharmaceutical Formulations Using Computational Tools. Braz. Arch. Biol. Technol..

[37] Zeng J., Gao H., Huang K., Tian X., Wang Z. (2022). Effects of Different Storage Temperatures on the Structure and Physicochemical Properties of Starch in Frozen Non-Fermented Dough. Food Sci. Technol..

[38] Li A., Gong T., Li X., Li X., Yang X., Guo Y. (2020). Preparation of Thermally Stable Emulsion Gels Based on Glucono-δ-Lactone Induced Gelation of Gellan Gum. Int. J. Biol. Macromol..

[39] Morris E.R., Nishinari K., Rinaudo M. (2012). Gelation of Gellan—A Review. Food Hydrocoll..

[40] Buldo P., Benfeldt C., Carey J.P., Folkenberg D.M., Jensen H.B., Sieuwerts S., Vlachvei K., Ipsen R. (2016). Interactions of Milk Proteins with Low and High Acyl Gellan: Effect on Microstructure and Textural Properties of Acidified Milk. Food Hydrocoll..

